# Integral Algorithms to Evaluate TiO_2_ and N-TiO_2_ Thin Films’ Cytocompatibility

**DOI:** 10.3390/ijms232315183

**Published:** 2022-12-02

**Authors:** Irina Yu. Zhuravleva, Maria A. Surovtseva, Alina A. Alshevskaya, Nikolay V. Surovtsev, Konstantin A. Okotrub, Irina I. Kim, Dmitriy A. Nasimov, Natalia A. Bondarenko, Oleg S. Kuzmin, Olga V. Poveshchenko

**Affiliations:** 1 E. Meshalkin National Medical Research Center of the RF Ministry of Health, 15 Rechkunovskaya St., 630055 Novosibirsk, Russia; 2 Research Institute of Clinical and Experimental Lymphology, Branch of the Federal Research Center Institute of Cytology and Genetics SB RAS, 2 Timakova St., 630060 Novosibirsk, Russia; 3 Biostatistics and Clinical Trials Center, Lavrentieva 6/1, 630090 Novosibirsk, Russia; 4 Federal State Budgetary Scientific Institution “Research Institute of Fundamental and Clinical Immunology” (RIFCI), 14, Yadrintsevskaya St., 630099 Novosibirsk, Russia; 5 Institute of Automation and Electrometry, Russian Academy of Sciences, 1 Academician Koptyug St., 630090 Novosibirsk, Russia; 6 Rzhanov Institute of Semiconductor Physics SB RAS, 30 Pirogova St., 630090 Novosibirsk, Russia; 7 Institute of Strength Physics and Materials Science of Siberian Branch Russian Academy of Sciences, 2/4, pr. Akademicheskii, 634055 Tomsk, Russia; 8 VIP Technologies Ltd., 634055 Tomsk, Russia

**Keywords:** titanium oxynitride, reactive magnetron sputtering, cytocompatibility, integral index, decision tree algorithm

## Abstract

Titanium oxide (TiO_2_) and oxynitride (N-TiO_2_) coatings can increase nitinol stents’ cytocompatibility with endothelial cells. Methods of TiO_2_ and N-TiO_2_ sputtering and cytocompatibility assessments vary significantly among different research groups, making it difficult to compare results. The aim of this work was to develop an integral cytocompatibility index (ICI) and a decision tree algorithm (DTA) using the “EA.hy926 cell/TiO_2_ or N-TiO_2_ coating” model and to determine the optimal cytocompatible coating. Magnetron sputtering was performed in a reaction gas medium with various N_2_:O_2_ ratios and bias voltages. The samples’ morphology was studied by scanning electron microscopy (SEM) and Raman spectroscopy. The cytocompatibility of the coatings was evaluated in terms of their cytotoxicity, adhesion, viability, and NO production. The ICI and DTA were developed to assess the cytocompatibility of the samples. Both algorithms demonstrated the best cytocompatibility for the sample sputtered at U_bias_ = 0 V and a gas ratio of N_2_:O_2_ = 2:1, in which the rutile phase dominated. The DTA provided more detailed information about the cytocompatibility, which depended on the sputtering mode, surface morphology, and crystalline phase. The proposed mathematical models relate the cytocompatibility and the studied physical characteristics.

## 1. Introduction

Titanium oxide (TiO_2)_ thin films have been known since the 1970s [1], but the first publications demonstrating the positive biomedical effects of TiO_2_ began to appear in the 1990s. It has been shown that TiO_2_ films cause an antibacterial effect [2], promote the formation of hydroxyapatite [3], reduce the corrosion of implants [4], etc. It was also found that the TiO_2_ layer of the rutile crystalline phase significantly increases the hemocompatibility of implants contacting the blood [5,6,7,8] and their cytocompatibility with endothelial progenitor cells [9]. This stimulated the development of endovascular devices coated with TiO_2_ [10]. Later, it turned out that even better biocompatibility is provided by N-doped TiO_2_ (N- TiO_2_) coatings [11]. It has been confirmed that TiO_x_N_y_-coated stents are less likely to undergo thrombosis and restenosis, even compared with limus-coated stents in clinical studies [12,13,14,15,16,17].

In the last decade, several studies have addressed the interaction between TiO_x_N_y_-coated surfaces and various types of cells: smooth muscle cells (SMC) [18], mesenchymal stem cells (MSC) [19], and endothelial cells (EC) [20]. It has been shown [19,20] that the cytocompatibility of a surface coated with N-doped TiO_2_ depends on the nitrogen content of the coating. The nanoroughness, hydrophilicity, crystallinity, and composition of the surface can be changed by selecting the gas composition of the reaction mixture, bias voltage, and the magnetron’s sputtering time [20,21]. These surface parameters influence the production of nitric oxide (NO) by endothelial cells and the cells’ adhesion, spreading, and viability on coated substrates. It is known that different cell lines react differently to an increase in the nitrogen content in the coating, which, for example, inhibits the proliferation and differentiation of MSCs [19], but improves the viability and NO production of endothelial cells [20].

The choice of composition for coating blood-contacting devices aims to achieve the desired effect. In the case of N-TiO_2_ coatings of intravascular and valvular stents, the goal is the “most favored” environment for endothelial cells and their progenitors (EPC) in order to accelerate and maximize the stents’ endothelialization. At the same time, it is desirable to suppress the growth and proliferation of SMC and fibroblasts, and reduce the proliferation and differentiation of MSCs. Coatings with such properties can significantly inhibit neointimal hyperplasia and the development of intravascular stent stenosis. For transcatheter valve stents, it is necessary to prevent the overgrowth of connective tissue and thrombosis on uncovered areas of the stent.

Methods for assessing cellular functions can vary significantly among research groups, which makes it difficult to compare their results quantitatively. In this regard, a unified approach is needed to assess the cytocompatibility of TiO_2_ and N-TiO_2_ coatings. This approach should be based on the unification of the results obtained by different biological tests. Since there are many variable parameters among the N-TiO_2_ coating methods, characterizing the structure and the composition of the coating by physical and chemical methods helps us to understand how and why thin films affect the cell–surface interaction. This understanding will allow regulation of the cellular response by changing the properties of the coating.

In addition, the application of a large set of biological, physical, and other tests to various coated surfaces requires a protocol for analyzing their joint data. The best way to do this is unclear. We previously faced this problem when studying six coatings of a nitinol substrate by using four biological and four physical tests [20].

The aim of this work was to develop an integral cytocompatibility index (ICI) and a decision tree algorithm (DTA) for assessing cytocompatibility using the EA.hy926 cell/TiO_2_ and N-TiO_2_ coating model, as well as to determine which of the studied coatings was optimally compatible with endothelial cells.

## 2. Results

### 2.1. Sample Characteristics

The gas ratio and bias voltage in the process of thin film sputtering varied (Table 1); however, the composition of the obtained samples mostly differed insignificantly (*p* > 0.05), which was showed by the results of the EDS analysis. The N concentration by mass fluctuated within 1%, the O concentration varied from 20.08% (min) to 24.36% (max). At a gas ratio of N_2_/O_2_ = 2:1 or 3:1 when sputtering, there was a slight tendency towards a decrease in the weight percentage of both N and O, if the sputtering was performed at U_bias_ = −100 V. The O/N atomic ratio in the thin films decreased slightly as the partial O_2_ pressure in the sputtering gas mixture decreased. However, this decrease did not match the O_2_/N_2_ ratio in the reactive plasma.

Based on these data, we considered that different partial gas pressures and bias voltage while sputtering did not affect the composition of thin films so much as their structure. This was confirmed by the results presented below.

### 2.2. Surface Characteristics

**Figure 1:**
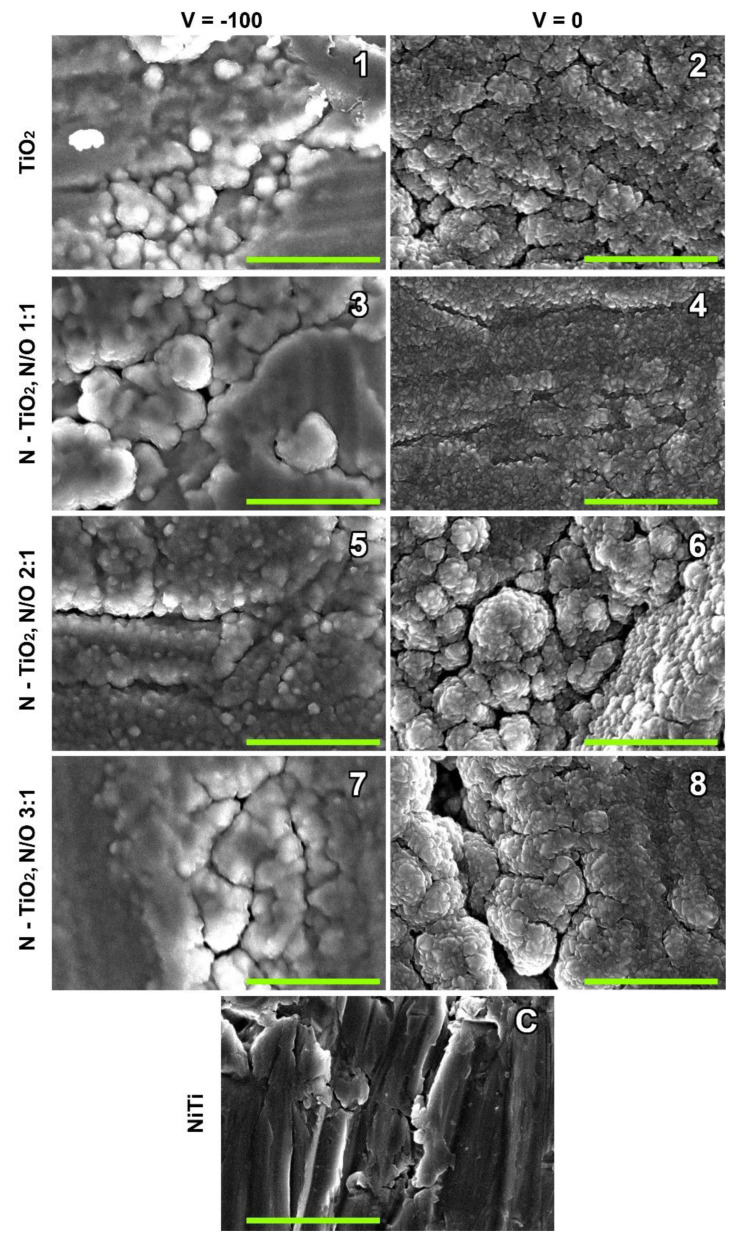
Samples Nos. 1–8 are TiO_2_- or TiO_x_N_y_-coated NiTi surfaces (the numbering is identical to that in Table 1). Sputtering was carried out at −100 V (left column) or 0 V (right column). The bottom image (**c**) presents the bare nitinol surface. Scale bars are 1 μm.

It was proven by the SEM that TiO_2_ and TiO_x_N_y_ thin films formed under all deposition modes (Figure 1). The micro- and nanostructures of the surfaces were significantly different in the three groups of samples. Bare nitinol and coatings formed at a bias voltage of 0 V and −100 V.

The surface of bare NiTi showed grooves that formed as a result of abrading. This observation is consistent with the data obtained by us earlier by atomic force microscopy [20].

In the case of the bias voltage of −100 V, the nanostructure of the obtained surfaces was quite amorphous, covered with cauliflower-like particles, and individual crystals were almost impossible to differentiate. Sample No. 5 (sputtered at a gas ratio of N_2_:O_2_ = 2:1) was somewhat different from the others. It showed large layers of an amorphous film with cracks; on the surfaces of the layers, spherical grains with a loss of crystallinity were visible, mainly 70–80 nm in size. The surface amorphization of other samples of this group (Nos. 1, 3, and 7) was even greater, and large conglomerates about 1 µm in diameter appeared.

Sputtering at 0 V (Nos. 2, 4, 6, and 8) resulted in surfaces with good crystallinity [19,20,21]. Samples No. 4, coated at the plasma gas ratio of N_2_:O_2_ = 1:1, demonstrated the highest ordering and low variability of crystal from 20 to 50 nm in size. In the other samples of this group, the sizes of the surface particles varied more widely, from 20 to 80 nm; at the gas ratio of N_2_:O_2_ = 3:1, they varied from 10 to 100 nm. The tendency of large crystalline conglomerates forming increased with an increasing nitrogen concentration.

### 2.3. Raman Scattering

**Figure 2:**
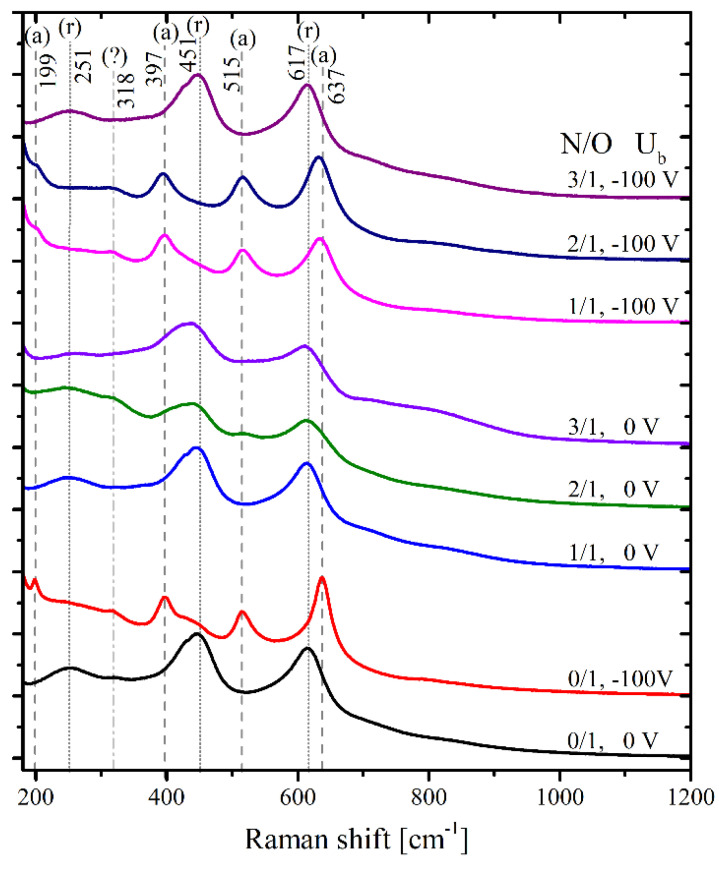
Raman spectra of the NiTi surfaces. Order of the spectra (from bottom to top): (1) N/O = 0/1, U = 0 V; (2) N/O = 0/1, U = −100 V; (3) N/O = 1/1, U = 0 V; (4) N/O = 2/1, U = 0 V; (5) N/O = 3/1, U = 0 V; (6) N/O = 1/1, U = −100 V; (7) N/O = 2/1, U = −100 V; (8) N/O = 3/1, U = −100 V. The vertical dashed lines indicate the Raman lines of anatase (a); the dotted lines denote the Raman lines of rutile (r). Spectra have been vertically shifted for clarity.

Surfaces were analyzed by using Raman spectroscopy (Figure 2). The main lines observed in the Raman spectra were assigned to rutile (250, 451, and 618 cm^−1^) and anatase (199, 396, 515, and 637 cm^−1^). Additional broad bands at ~715 and 800 cm^−1^ were suggested to be related to the combined modes. The Raman spectra indicated that the bias voltage increased the fraction of the anatase phase. For coatings formed at Ub = 0 V without nitrogen (N/O = 0/1), the Raman spectrum of pure rutile was observed. At Ub = −100 V, the spectrum was almost a pure anatase phase with a small admixture of rutile, as indicated by the related shoulder at 451 cm^−1^. This is consistent with earlier observations using X-ray diffraction for films synthesized with various Ub [21].

The addition of nitrogen to N/O = 1/1 caused minimal changes in the Raman spectra of oxide films. The only effect was a slight shift and broadening of the Raman lines for a film with Ub = −100 V. This effect indicates the increased concentration of defects and/or a decrease in the size of anatase crystallites. For coatings formed at Ub = 0 V, a further increase in the N/O ratio shifted the apparent maximum of the rutile Raman line from 451 cm^−1^ (N/O = 0/1) to 448 cm^−1^ at N/O = 2/1 and 445 cm^−1^ at N/O = 3/1. It is worth noting that this peak had a shoulder at ~425 cm^−1^, which may be related to the contribution of the rutile fraction with a non-stoichiometric O/Ti ratio of ~1.9 [22]. The second line at 618 cm^−1^ shows a maximum frequency shift of only 3 cm^−1^ (at N/O = 3/1), which indicates the characteristic size of rutile crystallites of at least 20 nm [23]. Along with the line shift, increases in the background intensity of the Raman crystalline lines and high-frequency overtones were observed. This observation indicated a violation of the selection rules for Raman scattering, which may be associated with a small crystallite size and/or an increased concentration of local defects in the oxide crystals. For coatings formed at Ub = −100 V, an increase in the N/O ratio gradually shifted the apparent maximum of the peak from 637.4 cm^−1^ at N/O = 0/1 to 634.8 cm^−1^ at N/O = 1/1, and 632 cm^−1^ at N/O = 2/1. Together with the frequency shift, this peak shows an increase in the width from 40 to 55 cm^−1^. The observed redshift and broadening of the peak at 637.4 cm^−1^ indicated a decrease in the size of the crystallites to about 10 µm [24]. A similar increase in width could be noted for the peak at 199 cm^-1^. In general, the Raman data showed that an increase in the N/O ratio increased the number of defects in NiTi oxide coatings.

At N/O = 3/1, the composition of the oxide changed abruptly from the predominance of anatase to rutile. It was interesting to note that the Raman spectrum of the coating formed at Ub = −100 V and N/O = 3 was much closer to the spectrum of the coating formed without nitrogen at Ub = 0 than that of the one at N/O = 3/1.

### 2.4. TiO_2_ and TiO_x_N_y_ Coatings’ Cytocompatibility

The cytotoxicity test results, adhesion, and viability of the EA.hy926 cells on the samples’ surfaces, as well as the production of NO, are presented in Table 2.

We scored each sample for each criterion using Table 3 and calculated mean scores for each group of samples (Tables S1–S4). The results are presented in Table 4. The ICI was calculated according to Equation (2) for each sample group. Samples No. 4 and No. 7, respectively, showed the highest ICI values of 6.2 and 5.8 points; sample No. 8 scored slightly lower (ICI = 5.1). All these samples were referred to as the group of optimal coatings.

Samples No. 2 and No. 5 had ICI scores of 3.9 and 3.2, respectively, which allowed them to be classified as acceptable coatings.

The lowest ICI (a score of 1.5) was, as expected, shown by the negative control (bare NiTi). Samples No.1 (a score of 1.6), No. 6 (a score of 1.8), and No. 3 (a score of 2.9) were also recognized as unacceptable.

**Figure 3:**
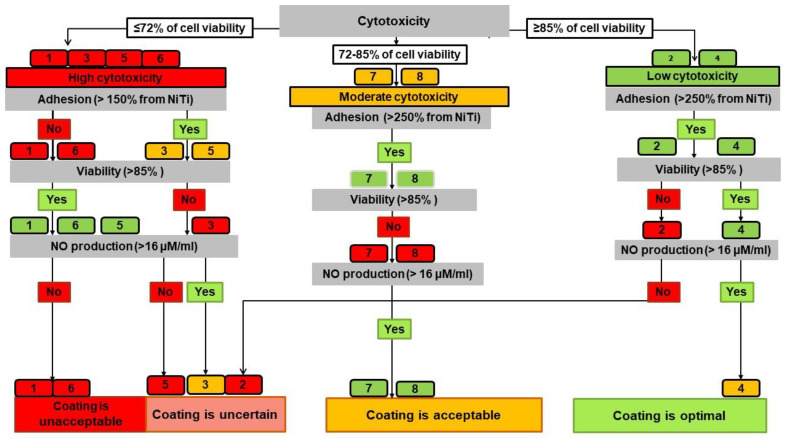
Decision tree algorithm for the cytocompatibility of TiO_2_- and N-TiO_2_-coatings.

An algorithm for selecting the optimal TiO_2_ and TiO_x_N_y_ coatings based on the cumulative evaluation of the measured indicators (Table 2) is presented as a decision tree with a hierarchical sequential assessment of the significance of each biological test (Figure 3).

Since direct cytotoxicity was considered the main criterion, the primary distribution into groups (according to Table 3) was as follows: Samples 2 and 4 were the least cytotoxic; Samples 7 and 8 had moderate cytotoxicity; and Samples 1, 3, 5, and 6 were more cytotoxic than bare nitinol. These results call into question the possibility of adequate engrafting and normal cell functioning for the last four samples. Further testing reinforced these doubts. Low (Nos. 1 and 6) or moderate (Nos. 3 and 5) adhesion and poor viability (No. 3) of the EA.hy926 cells were observed for these samples. Despite the high viability for Samples 1, 5, and 6, their NO-producing function was worse compared with cells contacting the bare NiTi. As a result, all coatings demonstrating high direct cytotoxicity formed the backbone of the “unacceptable” and “uncertain” groups, according to the specific evaluation parameters of the decision tree algorithm.

The most cytocompatible (by direct cytotoxicity test) coatings, Nos. 2 and 4, provided good cell adhesion, and No. 4 showed high viability and functional activity of the cells, in addition. However, thin film No. 2 (TiO_2_, U_bias_ = 0 V) demonstrated poor viability of the EA.hy926 cells and lower NO production than that of bare NiTi. This sample joined the “uncertain” group.

Thin films Nos. 7 and 8 formed at a gas ratio of nitrogen: oxygen = 3:1 showed very interesting features. They had different surface nanomorphologies (Figure 1), but the predominant TiO_2_ phase in both was rutile (Figure 2). All their cytocompatibility parameters were very close (Tables S5–S8): moderate direct cytotoxicity, high adhesion, and extremely high NO production, but their viability did not reach 85%. These coatings were classified as “acceptable”.

## 3. Discussion

Integral score assessment is widely used in biomedicine, as it allows us not only to refine prognostic and diagnostic models, but also to improve decision-making systems [25,26]. Integral indicators are important when it is necessary to evaluate and compare data from experiments of different types. The establishment of correlations and associations with the measured indicators of predictive outcomes (for example, [27]) can serve as a confirmation of the significance of such indicators and scores. Another way is to justify the significance of each of the components included in the model.

Data mining techniques can be classified into supervised (classification, regression) and unsupervised (clustering, association rules, correlations). Classification techniques are widely utilized [28]. Visualization of a decision-making algorithm in the form of a decision tree is one of the most widely used classification methods in data analysis [29], which has long been used in biology and medicine [30,31]. By using a decision tree, one can create models forecasting the outcome. In our case, it was to predict whether a NiTi stent with one coating or another would be compatible with endothelial cells.

Here, our task was to rank the cytocompatibility of TiO_2_ and TiO_x_N_y_ thin films sputtered on a NiTi surface under various conditions, as well as to assess their prospects for further research, including their cytocompatibility with various cell types. We chose two mathematical models for this purpose, namely ICI and a decision tree algorithm (DTA). These models allow one not only to compare different samples with each other, but also to understand which coating features affect the cell–surface interaction.

The development of ICI included the quantification of features characterizing the functional status of endothelial cells which are in contact with the TiO_2_ and TiO_x_N_y_ coatings, and the construction of a one-dimensional functional representation of their values. The cytocompatibility of the coatings was considered as an integrated system of characteristics obtained by biological tests for each sample.

When developing the models, we accounted for the concept that endothelial cells are the main cells of the endocardium and vascular intima; therefore, an increase in the stent’s endothelization would increase its hemocompatibility [32]. Endothelization can occur only due to the active adhesion of endothelial cells on the stent, maintaining their viability and functional activity (proliferation, migration, NO and cytokine production, etc.), which are directly related to the cytotoxicity of the stent material. Low cytotoxicity is the most important characteristic of an implant [33]; therefore, we chose this as the main criterion for a decision tree (DTA).

According to the ICI level and the decision tree, bare samples and TiO_2_- or TiO_x_N_y_-coated NiTi samples were divided into four groups with optimal, acceptable, uncertain and unacceptable cytocompatibility. The final results of the two solution algorithms generally did not contradict each other (Table 4), but the decision tree allowed us to perform more detailed differentiation of the surface cytocompatibility and hypothesize the mechanisms behind its variations. Thus, at the very beginning, when we evaluated the direct cytotoxicity, it was obvious that sputtering at −100V provides the most toxic thin films (Samples 1, 3, and 5). According to the SEM data, there was no distinct nanocrystallinity in these films, which are amorphous. The Raman spectra showed the predominance of the anatase phase in these thin films. However, the surface of No. 6 (N/O = 2/1, 0V) also fell into the cytotoxic group, although it had good crystallinity and a nanostructure similar to the other samples sputtered at 0 V (Figure 1). The Raman spectra of samples coated at 0 V are peculiar, with a shift in the maximum of the rutile line from 451 cm^−1^ (N/O = 0/1) to 448 cm^−1^, and with a weak contribution of anatase at 515 cm^−1^. At the same time, Sample 7, which had the highest N content (N/O = 3/1) and an amorphous surface, similar to the other films sputtered at −100 V, demonstrated the predominance of rutile, similar to Samples 2, 4, and 8, which is consistent with previous data [34]. Samples 2 and 4 were the best in terms of cytotoxicity, and thin films Nos. 7 and 8 had a moderate (acceptable) negative effect on EA.hy926 cells. In this regard, we hypothesized that the main contribution to the cytotoxicity of TiO_2_ and N-TiO_2_ films was made by the phase of oxide crystallites, and the predominance of rutile increased the cytocompatibility. The ordered crystalline nanostructure of the surface formed at 0 V also made a positive contribution, since the least cytotoxic were Samples 2 and 4, which differed not only in the high content of rutile, but also in the uniformity of the size and the arrangement of the crystals.

Our results are in good agreement with published data. It is known that TiO_2_ thin films are usually represented by anatase or rutile crystalline phases [35]. Rutile has previously been shown to have higher structural stability than anatase [36,37]. Rutile coatings can improve the biocompatibility of implants—[38,39]. Anatase, in contrast to rutile, is more cytotoxic, mainly due to the formation of reactive oxygen species [40].

All thin films with high direct cytotoxicity inhibit EA.hy926 cells’ adhesion (Figure 3). These results lead to a definite conclusion that the low adhesion is due solely to the high cytotoxicity of the material itself.

Sample No. 4 (U = 0V; N_2_/O_2_ = 1:1) had the optimal surface with the highest ICI (Table 4) and no undesirable feature (Figure 3). Acceptable cytocompatibility was demonstrated by Samples 7 and 8 because of their low cell viability but high NO production. Their ICI scores were in the range of 5.1–5.8, with moderate cytotoxicity and high adhesion. We hypothesized that the thin film covering these samples was capable of releasing NO by itself. Nitrogen likely acquires some lability in the crystal lattice at a high content in the gas reaction mixture during sputtering, and it can facilitate the release of NO into the conditioned medium when interacting with cells. The 3–3.5-fold higher NO level in the conditioned medium for Samples 7 and 8 compared with the control could not be attributed solely to the functional activity of the cells. Moreover, the cells’ viability on these surfaces was low.

The positive effects of nitrosation of an implant’s surface has been repeatedly shown, which include reduced late apoptosis of endothelial cells on the surface of 316L stainless steel [41], and the increased adhesion and proliferation of osteoblasts [42]. The nitrogen contained in the coating promotes the attachment, growth, and migration of endothelial cells and can potentially prevent platelet adhesion [43]. On the other hand, it is well known that an excessive paracellular NO concentration leads to a pronounced cytotoxic effect caused by the imbalance between oxidation and nitrosation [44]. As a result, the parameters of direct cytotoxicity and the relative content of living cells worsened in Samples 7 and 8.

We believe that the decision tree algorithm can successfully assess the cytocompatibility of coatings, because it visualizes certain patterns and covers the causal relationships between the tested criteria. In particular, the endothelial cells’ adhesion is directly related to the material’s cytotoxicity. In turn, the direct cytotoxicity of TiO_2_ and N-TiO_2_ coatings is closely related to the predominance of the rutile or anatase crystalline phase. Rutile-predominant surfaces are less cytotoxic (Samples 2, 4, 7, and 8). If the prevailing rutile is combined with ordered nanoroughness, then the cytotoxicity is minimal (Samples 2 and 4). Both samples were sputtered at 0 V and had a similar nanostructure, but Surface 4 contained nitrogen (N:O = 1:1), while Surface 2 did not. Low cell viability and NO production were observed for the TiO_2_ thin film (No. 2). Thus, we see that nitrogen doping improved the surface’s cytocompatibility, but an endotheliotoxic effect with low viability could be obtained with an excess of nitrogen (Nos. 7 and 8), although adhesion was the highest on these samples. Amorphous surfaces dominated by anatase (Nos. 1, 3, and 5) were characterized by high direct cytotoxicity, low adhesion, and low NO production by the EA.hy926 cells. All of them were classified as “unacceptable” or “uncertain”.

Some inconsistencies between the algorithms used should be noted. In particular, samples with optimal ICI values were divided by the DTA into optimal (No. 4) and acceptable (Nos. 7 and 8). The ICI ranked Samples 7 and 8 as optimal because of the high scores for NO production, although their viability was unsatisfactory. Similarly, the “acceptable” Nos. 2 and 5, according to the ICI, were classed as “uncertain” by DTA. Only Samples 1 and 6 were in the group of “unacceptable” in both algorithms.

Thus, the mathematical models considered here take all the studied criteria of cytocompatibility into account, determine the most promising types of coatings, and reveal the relationship between specific cytocompatibility parameters and the studied physical characteristics. This approach may be applicable for various coatings in order to make a justified decision about their cytocompatibility.

The developed algorithms can be used by researchers to assess the cytocompatibility of any surfaces of implantable medical devices, since any number of analyzed parameters can be entered into the integral indicator and into the decision tree, and threshold values can be set for each of the parameters. Additionally, any cells can be used to obtain data through experiments.

## 4. Materials and Methods

### 4.1. Sample Coatings

Flat samples of nitinol (NiTi, Ni 55.8%, Confluent Medical Technologies, Fremont, CA, USA) (8 × 8 × 0.5 mm) were used as the substrates. All the samples’ surfaces were sequentially abraded with a paper-backed SiC abrasive with crystal sizes P600, P1000, and P1500 (ISO 6344) and then cleaned with alcohol in an ultrasonic bath. The samples were coated by reactive magnetron sputtering of titanium in a reaction gas medium with various N_2_:O_2_ ratios (Table 5) using a TION-2M setup (Figure S1) developed by VIP technologies Ltd. (Tomsk, Russia) [21]. The fixed parameters of sputtering were a magnetron discharge power of 3 kW, a dual-system operating frequency of 40 kHz, a plasma-forming gas (argon) pressure o 0.065 Pa, and an exposure time of 45 min.

### 4.2. Scanning Electron Microscopy (SEM)

The surface morphology of the samples was analyzed using a high-resolution scanning electron microscope (Hitachi SU8220) with a cold field emission (CFE) gun and an upper detector filtering system to improve the selectivity of electron detection. Fine contrast filtering was achieved by selective filtering of inelastically scattered electrons and direct backscattered electrons with a certain level of energy. The samples were visualized at an accelerating voltage of 5 kV and a working distance of 9 mm, and with an upper secondary electron (SE) detector at various magnifications: ×1000, ×5000, ×10,000, ×20,000, and ×50,000.

EDS analysis of the thin films was carried out using a SU1000 FlexSEM II scanning electron microscope (Hitachi, Tokyo, Japan) equipped with an EDX system AzTec One (Oxford Instruments, Oxford, UK). Sample visualization was performed using SE detection with an electron beam of 15 kV energy and a high vacuum mode (Figure S2).

### 4.3. Raman Scattering

Raman spectra were measured using an in-lab built Raman setup composed of a microscope (Orthoplan, Leitz Wetzlar, Germany) and an imaging monochromator (SP2500i, Princeton Instruments) equipped with a LN-cooled CCD Spec-10:2K (Princeton Instruments). A solid-state laser Excelsior (Spectra-Physics) was used for Raman scattering excitation. Laser radiation at a wavelength of 532.1 nm and 5 mW of power was focused on the sample on a spot measuring ~1 µm^2^. Backscattered light was collected using the microscope’s objective (PL FLUOTARL, Leica Microsystems) and analyzed with a spectrometer. The spectral resolution was 2.5 cm^−1^.

For each sample studied, the Raman spectra from at least five random local areas were measured and averaged. Preliminary spectral analysis included intensity burst correction and baseline subtraction via a linear function. The spectra’s wavenumbers were calibrated by the spectrum of a neon discharge lamp. The apparent frequencies of the Raman lines were evaluated from the second derivative analysis.

### 4.4. Cytocompatibility Evaluation

#### 4.4.1. Materials

NiTi samples coated with TiO_2_ or TiO_x_N_y_ were sterilized by 30 min of incubation in 70 vol% aqueous ethanol and then washed three times with sterile phosphate-buffered saline (PBS). The bare NiTi samples were the control group.

The EA.hy926 endothelial cells were kindly provided by Dr. C. J. Edgell (Carolina University, USA). The EA.hy926 cells were grown in DMEM/F12 (Gibco, Carlsbad, CA, USA) medium supplemented with 10% fetal calf serum (FCS; Hyclone, Logan, UT, USA), 40 μg mL^−1^ gentamicin sulfate (Dalkhimpharm, Khabarovsk, Russia), and 2 mM L-glutamine (ICN, Costa Mesa, CA, USA) in a humid atmosphere with 5 vol% CO_2_ at 37 °C until a confluent monolayer was formed. The cells were cultured in flasks and removed with Trypsin-Versene (Biolot, Saint Petersburg, Russia) during passaging.

#### 4.4.2. Direct Cytotoxicity Evaluation

The EA.hy926 cells were seeded in a 24-well cell culture plate at 4 × 10^4^ cells per well and incubated for 24 h to allow attachment. The bare NiTi and eight coated samples were directly deposited over cell cultures in each well. Glass coverslips were used as a cytocompatible control material. After 72 h of cultivation, all samples were removed. Cell viability was then determined using an MTT assay (Sigma-Aldrich, Darmstadt, Germany) according to the manufacturer’s instructions. MTT (10 μL, 5 mg mL^−1^) was added to each well, and the incubation was continued for another 4 h. Formazan crystals that formed after 4 h in each well were dissolved in dimethyl sulfoxide (150 μL; PanReac AppliChem, Darmstadt, Germany). The absorbance of the dissolved formazan crystals was measured at 492 nm using a Stat Fax-2100 multiwall plate reader (Awareness Technology, Inc., Palm City, FL, USA). Cell viability was calculated using the equation

Viability (%) = OD_experimental group_/OD_control group_) × 100 (1)

where OD is the optical density of the samples, and the positive control group was cells cultivated with glass coverslips.

#### 4.4.3. Evaluation of Cell Adhesion 

The EA.hy926 cells were exposed to the surfaces of coated NiTi samples (5 × 10^4^ cells in 20 μL per sample), cultured for six days, and stained with phalloidin and 4′,6-diamidino-2-phenylindole (DAPI, Abcam, Cambridge, UK) to expose the actin cytoskeleton. Phalloidin was conjugated to Alexa Fluor 488 (Thermo Fisher Scientific, Waltham, MA, USA) and incubated at a dilution of 1:200 (in PBS) for 1 h according to the manufacturer’s instructions. The surfaces of the samples were then imaged using an Axio Observer microscope (Zeiss, Oberkochen, Germany). Cell quantification was performed using at least five microscopic images of each surface, and the results were expressed as the number of adhered cells per mm^2^.

#### 4.4.4. Cell Viability Assay

TiO_2_- and TiO_x_N_y_-coated samples were placed into the wells of a 24-well plate (one sample per well) and exposed to cells (5 × 10^4^ cells in 20 μL per sample), followed by cultivation for six days. The numbers of live and dead cells were determined by staining with fluorescent dyes, namely acridine orange (DIA M, Russia; 100 μg mL^−1^) and propidium iodide (Medigen, Novosibirsk, Russia; 100 μg mL^−1^), which stain live and dead cells, and only dead cells, respectively. The samples were then incubated for 10 min at 37 °C and examined using an Axio Observer microscope (Zeiss, Oberkochen, Germany). For the assay, at least 500 cells were counted.

#### 4.4.5. NO Production Assay

NO production was assessed by measuring the levels of nitrite as a stable end product using the Griess reagent (Sigma-Aldrich, Darmstadt, Germany) according to the manufacturer’s instructions. The EA.hy926 cells were seeded on the surface of the sample in the wells of a 24-well plate (5 × 10^4^ cells per sample). After 24 h of cultivation, 50 μL of the supernatant was harvested and 50 μL of the Griess reagent was added to a 96-well plate. The absorbance at 492 nm was measured using a microplate reader (Stat FAX-2100, Awareness Technology Inc., USA), and the nitrite concentrations were estimated using a standard calibration curve.

### 4.5. Development of an Algorithm for Assessing the Cytocompatibility of Coatings

We used two methods for assessing the total score of the thin films’ biocompatibility, taking all the parameters of the biological tests into account.

#### 4.5.1. Integrated Assessment of the Studied Indicators

The first method was to calculate the numerical integral cytocompatibility index (ICI).

The following key criteria were included in the total score assessment:

1. The direct cytotoxicity of thin films (by the MTT test) to assess the toxic effect of thin films which can prevent cell-to-surface contacts in living systems;
2. The adhesion of cells on the samples’ surface to assess the probability of cells seeding after contact;
3. Cell viability to assess the relative survival of cells on the samples’ surface, indicating the formation of a proliferating cell biofilm;
4. NO production to assess the functional activity of endothelial cells.

Limiting parameter ranges were converted to scores for each of these criteria (Table 3) as follows:

0 = unacceptable level, demonstrating no positive or negative effect of the coating;

1 = moderate range of values within acceptable but non-optimal levels;

2 = optimal level, which is the most favorable for cell engraftment and growth.

The parameter ranges were chosen on the basis of published data and our own experience with cell cultures.

When evaluating the direct cytotoxicity, we followed the ISO 10993-5 standard, slightly increasing the lower threshold limit (70% in the standard) [45]. Since we were trying to improve the biocompatibility of the substrate material with a thin film, it was advisable to start from 72% of the level of the negative control (bare NiTi). All values below or equal to this level were taken as unacceptable cytotoxicity. We considered the acceptable range to be values that were higher than those of the negative control, but not more than 85%. Our experience with cell cultures allowed us to assert that a rate of cell survival more than 85% is a good result.

A similar approach using a negative control was applied to the assessment of adhesion. All values less than 150% of the negative control were classified as unacceptable, those from 150% to 250% were taken as acceptable, and over 250% were classed as optimal.

Parameters for evaluating the cells’ viability were set to the average between the ISO 10993-5 standard (80% living cells) [45] and the clinical values (90%) applied to cell viability in vitro for 6 days [46]. If living cells accounted for 85%, the surface was considered to be cytocompatible; if this value was less than 85%, the surface was not cytocompatible.

The cells’ functional activity was assessed by NO production [44]. The mean NO level (<16 µM/mL) in the conditioned medium with bare NiTi samples was taken as a control; equal or lower levels were considered to be unacceptable, indicating the insufficient functional activity of the EA.hy926 cell culture on the surface of the samples. A level of NO production in the range of 100–150% (16–24 µM/mL) of the bare NiTi control’s level was considered to be acceptable and indicative of moderate functional activity. The NO content in the conditioned medium was over 24 µM/mL, which we considered to be the optimal level of NO production.

The cells’ functional activity was assessed by their NO production [44]. The mean NO level (<16 µM/mL) in the conditioned medium with the bare NiTi samples was taken as the control; equal or lower levels were considered to be unacceptable, indicating insufficient functional activity of the EA.hy926 cell culture on the surface of the samples. A level of NO production in the range of 100–150% (16–24 µM/mL) of the bare NiTi control’s level was considered to be acceptable and indicative of moderate functional activity. An NO content in the conditioned medium of over 24 µM/mL was considered to be the optimal level of NO production.

After we had obtained the results of the biological tests for each sample, scoring was performed for all criteria. The mean score of each criterion was determined for each group of samples. The integral cytocompatibility index (ICI) of each group was calculated as the sum of the mean scores:

ICI = Mean score (cytotoxicity) + Mean score (adhesion) + Mean score (viability) + Mean score (NO production)(2)

Therefore, the maximum possible ICI score was 8 and the minimum was 0. We classified the tested thin film samples as optimal when ICI was at least scores (≥60% of the maximum ICI), acceptable with a total score of 3.2–4.7 (40% < maximum ICI < 60%), and unacceptable when the ICI was less than 3.2 (<40% of the maximum ICI).

#### 4.5.2. Decision Tree Algorithm

As the second method for assessing the biocompatibility of TiO_2_ and TiO_x_N_y_ coatings, we chose the decision-tree-like graph visualization [30].

We used the previously defined boundary values as the branch node (Table 3) and the overall level of thin film cytocompatibility as the predicted outcome.

The level of direct cytotoxicity was taken as the main criterion of biological significance. Subsequent criteria, namely adhesion, viability, and NO production by endothelial cells on TiO_2_ and TiO_x_N_y_ samples, were taken to be equal. Each tree node represented the parameter range of the selected criterion. Each “leaf” (box) indicated the sample’s number and its class membership («yes» or «no»). The leaf’s color reflected the appropriate parameter levels: red was unacceptable, yellow was moderate, and green was optimal.

The final decision was substantiated by the following interpretation of the set of characteristics:

- Optimal cytocompatibility had a surface with no red leaf and at least two green leaves;
- A surface was considered acceptable with three green or yellow leaves;
- The cytocompatibility was classified as uncertain when two leaves were red;
- The surface was determined to be unacceptable when three leaves were red.

### 4.6. Statistical Analysis

Statistical analysis (Tables S1–S8) was carried out using STATISTICA 10.0 software (StatSoft, Tulsa, OK, USA). Statistical significance for all tests was defined as a *p*-value < 0.05. The quantitative data are presented as the mean (M) ± standard deviation (SD). Comparisons of samples were performed by analysis of variance (Kruskal–Wallis rank test) with multiple comparison of the medians.

## 5. Conclusions

1. By comparing two algorithms for assessing the thin films’ cytocompatibility, we have shown that DTA provides more detailed information on the changes in the properties of the studied coatings, which depend on the sputtering mode, the surface morphology, and the crystalline phase.
2. N-TiO_2_ coatings sputtered in modes providing a predominance of the rutile crystalline phase make it possible to obtain the most cytocompatible thin films.
3. The evaluation of thin films using both algorithms demonstrated the best cytocompatibility of Sample No. 4 sputtered at 0 V and N:O = 1:1. The surfaces of Samples No. 7 (−100 V; N:O = 3:1) and No. 8 (0 V; N:O = 3:1) had acceptable cytocompatibility.

## 6. Limitations

Cellular function was studied by using only NO production in this study. This cannot provide complete information on the potential effects of the coatings’ composition and morphology on cellular function. In addition, it is not clear what caused the high NO level of cells contacting the surfaces sputtered at N/O = 3/1, e.g., whether this was only caused by cellular function or the additional release of NO by the surfaces themselves. Further studies are necessary for studying the cellular function.

## Figures and Tables

**Table 1:** Sample characteristics.

Sample No.	O_2/_N_2_ Ratio	Bias Voltage, V	Film	N (wt%)	O (wt%)	O/N AR
	(While Sputtering)			In the Thin Film		
1	-	−100	TiO_2_	-	22.07 ± 0.09	-
2	-	0	TiO_2_	-	22.27 ± 0.12	-
3	1	−100	Ti-O-N	5.42 ± 0.28	24.36 ± 0.61	3.94 ± 0.14
4	1	0	Ti-O-N	5.13 ± 0.06	23.97 ± 0.23	4.08 ± 0.01
5	0.5	−100	Ti-O-N	4.87 ± 0.12	21.01 ± 0.20	3.79 ± 0.12
6	0.5	0	Ti-O-N	5.31 ± 0.08	22.95 ± 0.14	3.79 ± 0.04
7	0.33	−100	Ti-O-N	4.77 ± 0.05	20.08 ± 0.29	3.68 ± 0.09
8	0.33	0	Ti-O-N	4.85 ± 0.05	21.55 ± 0.05	3.89 ± 0.05
C	-	-	-	-	-	-

Abbreviations: wt%, element’s percentage by mass; AR, atomic ratio; C, control samples (bare NiTi).

**Table 2:** Cytocompatibility test results. Data (n = 6 in each group) are presented as M ± SD.

Sample No.	Direct Cytotoxicity, %	Adhesion and Spreading of Cells’ Relative Content from NiTi, %	Viability of Cells, %	NO Production, µM/mL
No. 1	63.9 ± 2.9	114.5 ± 23.3	87.4 ± 7.5	14.6 ± 3.1
No. 2	87.1 ± 3.4	261.9 ± 67.7	75.8 ± 9.2	13.5 ± 2.6
No. 3	66.9 ± 4.2	180.6 ± 60.9	83.0 ± 5.8	17.7 ± 0.5
No. 4	86.6 ± 5.3	279.5 ± 20.8	88.7 ± 3.3	22.1 ± 5.4
No. 5	68.8 ± 3.6	180.2 ± 19.0	94.4 ± 2.3	9.8 ± 3.2
No. 6	70.2 ± 4.5	126.4 ±34.2	87.5 ± 2.7	12.5 ± 2.4
No. 7	79.1 ± 3.1	312.1 ± 38.2	84.1 ± 4.9	56.2 ± 31.4
No. 8	79.0 ± 3.5	259.0 ± 39.1	83.1 ± 6.2	52.2 ± 13.7
Control (NiTi)	71.7 ± 3.6	100 ± 0	64.6 ± 29.7	16.1 ± 2.4

**Table 3:** Ranking the assessment of the biological test results.

Criterion	Parameter		
	Unacceptable Level (Score = 0)	Moderate Level (Score = 1)	Optimal Level (Score = 2)
Direct cytotoxicity, % (relative cell viability in the MTT test)	Bare NiTi control’s level or less (≤72%), high surface cytotoxicity	> Bare NiTi control ≤85% (73–85%), moderate cytotoxicity	>85%, low cytotoxicity
Cell adhesion, % (390 cells/mm^2^ = 100% adhesion on the bare NiTi surface)	<150% of the bare NiTi control’s level, low adhesion	150–250% of the bare NiTi control’s level, moderate adhesion	>250% of the bare NiTi control’s level, high adhesion
Cell viability, % (relative content of living cells on the sample’s surface)	<85%, non-cytocompatible surface	-	≥85%, cytocompatible surface
NO production, μM/mL, (16 μM/mL- NO production on the bare NiTi surface)	Bare NiTi control’s level or less (<16 μM/mL), low functional activity	>Bare NiTi control, but <150% of the bare NiTi control’s level (16–24 μM/mL), moderate functional activity	>150% of the bare NiTi control’s level (>24 μM/mL). high functional activity

**Table 4:** The cytocompatibility scores of the TiO_2_ and N-TiO_2_ thin films.

Criterion	No. 1	No. 2	No. 3	No. 4	No. 5	No. 6	No. 7	No. 8	Control (NiTi)
Direct cytotoxicity	0	1.8 ± 0.41	0.3 ± 0.52	1.5 ± 0.55	0.2 ± 0.41	0.3 ± 0.52	1 ± 0	1.2 ± 0.41	0.3 ± 0.52
Adhesion and spreading of cells	0	1.6 ± 0.55	0.8 ± 0.50	2 ± 0	1 ± 0	0.3 ± 0.50	2 ± 0	1.3 ± 0.50	0
Viability of cells	1.3 ± 0.82	0.5 ± 0.55	0.8 + 0.45	1.4 ± 0.55	2 ± 0	1.2 ± 0.45	0.8 ± 0.45	0.6 ± 0.89	0.7 ± 1.0
NO production	0.3 ± 0.52	0 ± 0	1 ± 0	1.3 ± 0.52	0 ± 0	0 ± 0	2 ± 0	2 ± 0	0.5 ± 0.55
ICI (ICI_max_ = 8)	1.6	3.9	2.9	6.2	3.2	1.8	5.8	5.1	1.5

**Table 5:** Details of the deposition modes used.

Sample No.	Partial Gas Pressure, Pa, N_2_/O_2_	Negative Bias Voltage	Films	N_2_/O_2_ Ratio
control (NiTi)	-	-	-	-
1	0/0.130	U = −100 V	TiO_2_	-
2	0/0.130	U = 0	TiO_2_	-
3	0.065/0.065	U = −100 V	Ti-O-N	1/1
4	0.065/0.065	U = 0	Ti-O-N	1/1
5	0.087/0.046	U = −100 V	Ti-O-N	2/1
6	0.087/0.046	U = 0	Ti-O-N	2/1
7	0.102/0.033	U = −100 V	Ti-O-N	3/1
8	0.102/0.033	U = 0	Ti-O-N	3/1

## Data Availability

Not applicable.

## Supplementary Materials

The supporting information can be downloaded at: https://www.mdpi.com/article/10.3390/ijms232315183/s1.
