# Supplementary material for: Integral Algorithms to Evaluate TiO2 and N-TiO2 Thin Films’ Cytocompatibility
Source: Int J Mol Sci. 2022 Dec 2;23(23):15183. doi: 10.3390/ijms232315183 (PMC9736632; doi:10.3390/ijms232315183)
Supplement: Supplementary file 1 [file ijms-23-15183-s001.zip › ijms-2006870-supplementary.pdf]

Supplementary

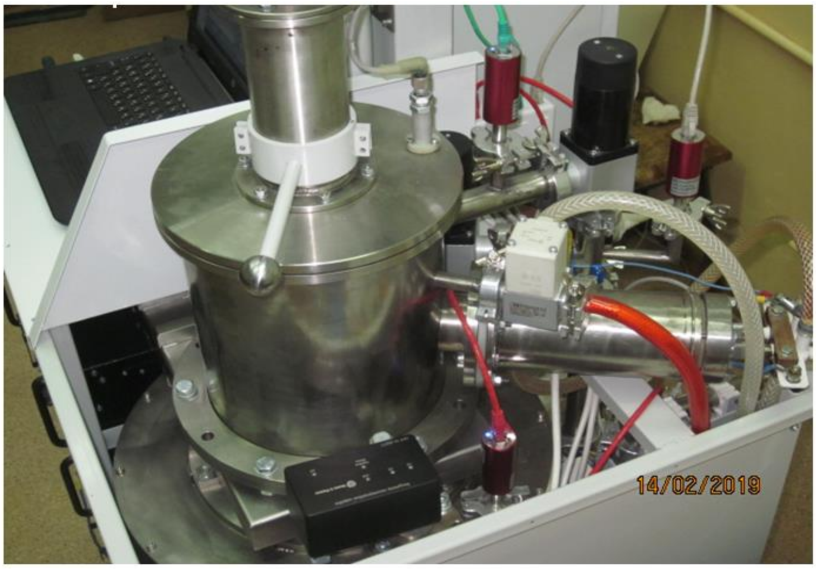

Figure. S1. TION-2M setup used for reactive magnetron sputtering (VIP technologies Ltd., Tomsk, Russia).

Table S1. Statistical significance of differences in direct cytotoxicity after 72-h direct contact with samples

Direct cytotoxicity, %	**No.1**	**No.2**	**No.3**	**No.4**	**No.5**	**No.6**	**No.7**	**No.8**	**control (NiTi)**
**No.1**		0.000	1.000	0.000	1.000	1.000	0.060	0.090	1.000
**No.2**	0.000		0.002	1.000	0.013	0.038	1.000	1.000	0.155
**No.3**	1.000	0.002		0.004	1.000	1.000	0.302	0.425	1.000
**No.4**	0.000	1.000	0.004		0.025	0.068	1.000	1.000	0.260
**No.5**	1.000	0.013	1.000	0.025		1.000	1.000	1.000	1.000
**No.6**	1.000	0.038	1.000	0.068	1.000		1.000	1.000	1.000
**No.7**	0.060	1.000	0.302	1.000	1.000	1.000		1.000	1.000
**No.8**	0.090	1.000	0.425	1.000	1.000	1.000	1.000		1.000
**control (NiTi)**	1.000	0.155	1.000	0.260	1.000	1.000	1.000	1.000	

Table S2. Statistical significance of differences of cell adhesion after six-day cultivation on sample surfaces

Adhesion and spreading of cells, cells per mm^2	**No.1**	**No.2**	**No.3**	**No.4**	**No.5**	**No.6**	**No.7**	**No.8**	**control (NiTi)**
**No.1**		0.184	1.000	0.056	1.000	1.000	0.011	0.188	1.000
**No.2**	0.184		1.000	1.000	1.000	0.955	1.000	1.000	0.023
**No.3**	1.000	1.000		1.000	1.000	1.000	0.509	1.000	1.000
**No.4**	0.056	1.000	1.000		1.000	0.338	1.000	1.000	0.006
**No.5**	1.000	1.000	1.000	1.000		1.000	0.911	1.000	1.000
**No.6**	1.000	0.955	1.000	0.338	1.000		0.088	0.813	1.000
**No.7**	0.011	1.000	0.509	1.000	0.911	0.088		1.000	0.001
**No.8**	0.188	1.000	1.000	1.000	1.000	0.813	1.000		0.033

control (NiTi)	1.000	0.023	1.000	0.006	1.000	1.000	0.001	0.033	

Table S3. Statistical significance of differences of cell viability after six-day cultivation on sample surfaces

Viability of cells, %	**No.1**	**No.2**	**No.3**	**No.4**	**No.5**	**No.6**	**No.7**	**No.8**	**control (NiTi)**
**No.1**		1.000	1.000	1.000	1.000	1.000	1.000	1.000	1.000
**No.2**	1.000		1.000	0.874	0.009	1.000	1.000	1.000	1.000
**No.3**	1.000	1.000		1.000	0.404	1.000	1.000	1.000	1.000
**No.4**	1.000	0.874	1.000		1.000	1.000	1.000	1.000	1.000
**No.5**	1.000	0.009	0.404	1.000		1.000	0.604	0.381	0.033
**No.6**	1.000	1.000	1.000	1.000	1.000		1.000	1.000	1.000
**No.7**	1.000	1.000	1.000	1.000	0.604	1.000		1.000	1.000
**No.8**	1.000	1.000	1.000	1.000	0.381	1.000	1.000		1.000
**control (NiTi)**	1.000	1.000	1.000	1.000	0.033	1.000	1.000	1.000	

Table S4. Statistical significance of differences of NO production by endothelial cells within 24 h

NO production, μM/mL	**No.1**	**No.2**	**No.3**	**No.4**	**No.5**	**No.6**	**No.7**	**No.8**	**control (NiTi)**
**No.1**		1.000	1.000	1.000	1.000	1.000	1.000	1.000	1.000
**No.2**	1.000		1.000	1.000	1.000	1.000	1.000	1.000	1.000
**No.3**	1.000	1.000		1.000	1.000	1.000	1.000	1.000	1.000
**No.4**	1.000	1.000	1.000		0.468	1.000	1.000	1.000	1.000
**No.5**	1.000	1.000	1.000	0.468		1.000	0.101	0.101	1.000
**No.6**	1.000	1.000	1.000	1.000	1.000		0.479	0.479	1.000
**No.7**	1.000	1.000	1.000	1.000	0.101	0.479		1.000	1.000
**No.8**	1.000	1.000	1.000	1.000	0.101	0.479	1.000		1.000
**control (NiTi)**	1.000	1.000	1.000	1.000	1.000	1.000	1.000	1.000	

Table S5. Scores assigned to each sample in a direct cytotoxicity test

sample number, (n)	**No.1**	**No.2**	**No.3**	**No.4**	**No.5**	**No.6**	**No.7**	**No.8**	**control (NiTi)**
1	0	2	1	2	1	1	1	2	1
2	0	1	0	1	0	0	1	1	0
3	0	2	0	2	0	0	1	1	0
4	0	2	0	1	0	0	1	1	0
5	0	2	0	1	0	0	1	1	0
6	0	2	1	2	0	1	1	1	1
Mean	0.0	1.8	0.3	1,5	0.2	0.3	1	1.2	0.3
SD	0.0	0.41	0.52	0.55	0.41	0.52	0.0	0.41	0.52

Table S6. Scores assigned to each sample in cell adhesion test

sample number, (n)	**No.1**	**No.2**	**No.3**	**No.4**	**No.5**	**No.6**	**No.7**	**No.8**	**control (NiTi)**
1	0	1	0	2	1	0	2	1	0
2	0	2	1	2	1	0	2	2	0
3	0	2	1	2	1	0	2	1	0
4	0	1	1	2	1	1	2	1	0
5	0	2		2	1				0
Mean	0	1.6	0.8	2	1	0.3	2	1.3	0
SD	0	0.55	0.50	0	0	0.50	0	0.50	0

Table S7. Scores assigned to each sample in the viability test

sample number, (n)	**No.1**	**No.2**	**No.3**	**No.4**	**No.5**	**No.6**	**No.7**	**No.8**	**control (NiTi)**
1	2	1	0	2	2	2	1	1	0
2	2	1	1	1	2	1	1	2	0
3	1	0	1	1	2	1	1	0	0
4	0	0	1	2	2	1	1	0	2
5	1	0	1	1	2	1	1	0	2
6	2	1					0		0
7									1
Mean	1.3	0.5	0.8	1.4	2	1.2	0.8	0.6	0.7
SD	0.82	0.55	0.45	0.55	0	0.45	0.45	0.89	1.0

Table S8. Scores assigned to each sample in the nitric oxide (NO) production test

sample number, (n)	**No.1**	**No.2**	**No.3**	**No.4**	**No.5**	**No.6**	**No.7**	**No.8**	**control (NiTi)**
1	1	0	1	1	0	0	2	2	0
2	0	0	1	1	0	0	2	2	1
3	0	0	1	2	0	0	2	2	0
4	0	0	1	1	0	0	2	2	0
5	0	0	1	2	0	0	2	2	1
6	1	1	1	1	0	0	2	2	1
Mean	0.3	0	1	1.3	0	0	2	2	0.5
SD	0.52	0	0	0.52	0	0	0	0	0.55

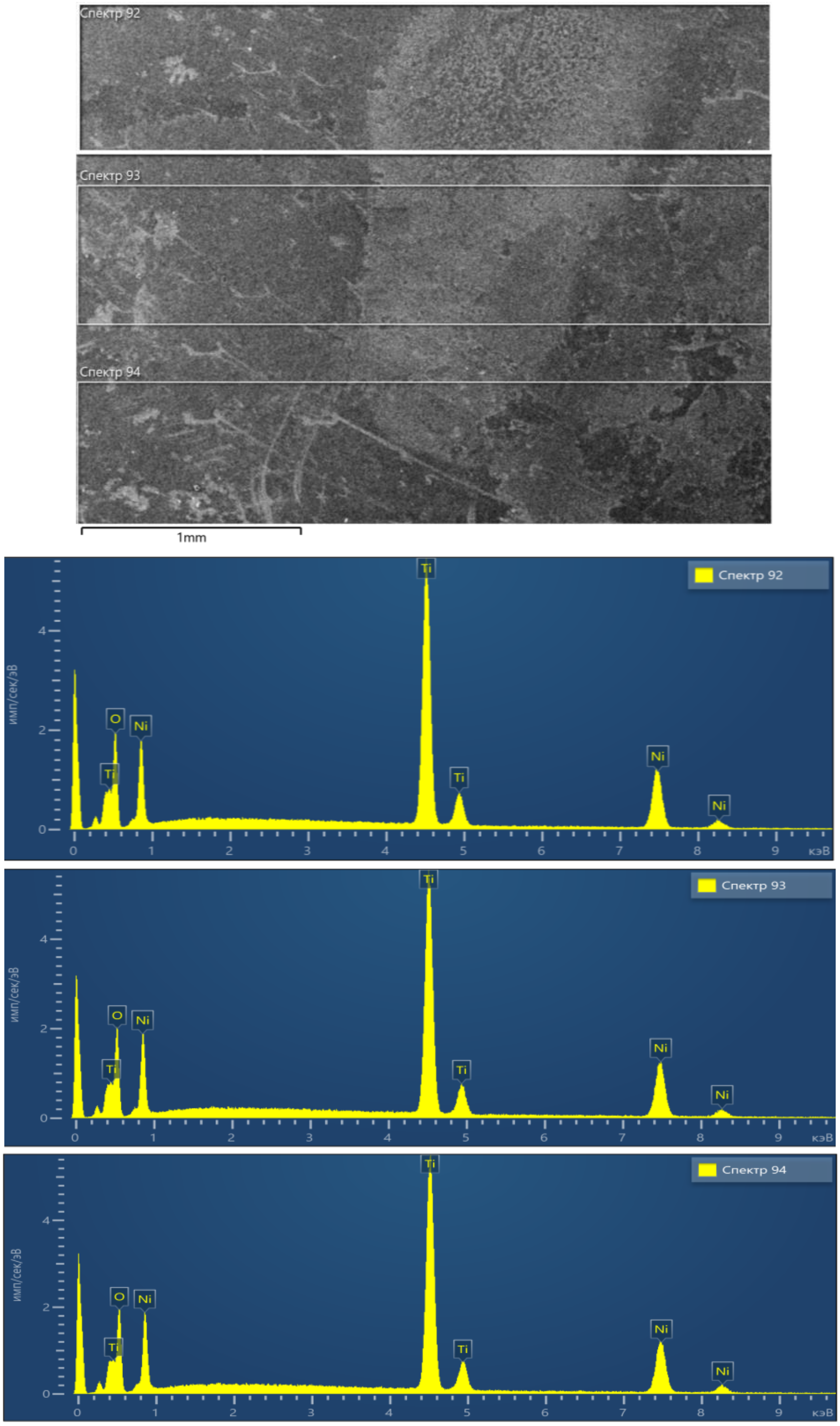

Figure S2. EDS analysis. Three spots selected on the surface of the sample No.1 (top image) and 3 element spectra obtained from these fields.
